# Hearing and vision difficulty and sequential treatment among older adults in India

**DOI:** 10.1038/s41598-022-21467-y

**Published:** 2022-11-09

**Authors:** Strong P. Marbaniang, Ratna Patel, Pradeep Kumar, Shekhar Chauhan, Shobhit Srivastava

**Affiliations:** 1 Department of Statistics, Sankardev College, Shillong, Meghalaya India; 2 Department of Public Health and Mortality Studies, International Institute for Population Sciences, Mumbai, India; 3 Department of Survey Research & Data Analytics, International Institute for Population Sciences, Mumbai, India; 4 Department of Family and Generations, International Institute for Population Sciences, Mumbai, India

**Keywords:** Diagnosis, Geriatrics

## Abstract

Aging not only affect biomarker-related processes, but it also affects the physiological processes of the human body. Of all the physiological processes, hearing and vision are of utmost importance to a human. Therefore, this study examines the prevalence and factors associated with hearing and vision difficulty and their sequential treatment among older adults in India. Utilizing data from Building a Knowledge Base on Population Aging in India, study used two sets of outcome variables; firstly, self-reported hearing and vision difficulty and secondly, treatment-seeking for hearing and vision difficulty. A total of 9541 older adults aged 60+ years from seven major regionally representative states were selected. Descriptive statistics were used to perform preliminary analysis. Additionally, the study employed the Heckprobit selection model. It is a two-equation model. This model is used in order to accommodate the heterogeneity (i.e., shared unobserved factors) among older adults and then address the endogeneity (between hearing and vision loss problems and their treatment-seeking behaviour) for older adults in India, the model offers a two-step analysis and deals with the zero-sample issue. Around 59% and 21% of older adults reported vision and hearing difficulty, respectively. Only 5% of older adults suffering from hearing difficulty reported utilizing hearing aids. Lifestyle factors (smoking tobacco and chewing tobacco) significantly affect hearing and vision difficulty; various chronic diseases were also found to be associated with high levels of hearing and vision difficulty among older adults. Results from Heckprobit model shows that older adults with 11+ years of education had higher probability to use visual [β = 0.54, 95% confidence interval (CI): 0.37, 0.70] and hearing aids [β = 0.6, 95% CI: 0.18, 1.02]. The use of hearing and vision aids was lower among poor older adults, older adults from Scheduled Caste, and older adults in rural areas. The study indicates that more than half of older adults face vision difficulty and almost one-fourth face hearing difficulty in rural India, education and lifestyle appear to be the main driver of health-seeking behaviour. Additional attention shall be given to understand the strategies that may advocate a higher use for hearing aids among older adults.

## Introduction

The etiology of the ageing process is a cumbersome process and is yet to be fully developed^1^. Various researcher have shared their views on the definition of ageing, and most of them agreed that ageing is the gradual accumulation of deleterious biological changes that are accompanied by a progressive loss of function^2,3^. The world is getting older faster than ever. With a decline in fertility and an increase in life expectancy around the globe, the ageing population is on the rise. This rise in ageing population is more worrisome in Europe and other western countries than anywhere else. In India, the ageing population has increased sharply during recent years^4^. The rise in ageing population is posing a challenge at the policy front. Researchers unanimously have associated aging with various degenerative problems^4^.

Aging not only affect biomarker related processes, but it also affects the physiological processes of the human body^5^. Hearing and vision difficulty are highly associated with ageing population across the populations^6–13^. Hearing and vision difficulty among older adults significantly affect their quality of life^6,14^. Hearing and vision difficulty is a common problem among older adults, and the impact of such loss may be profound^15,16^. The hearing and vision difficulty may have consequences for the social, functional, and psychological well-being of older adults^12,15–17^. Evidence from a large body of literature suggest a relationship of unhealthy behaviour such as smoking and drinking alcohol with the incidence of vision impairment^19,20^. A cohort study among the older adults conclude that participants with three unhealthy behaviour i.e., low die quality, heavy smoking and sedentary lifestyle increased the odds of vision impairment by threefold^21^. The biological mechanisms explain that smoking may reduce choroidal blood flow in the eye, and promote ischaemia, micro-infarctions, and hypoxia, all of which may increase the risk of vision impairment^22,23^. Also, there is a dirt of literatures on the direct relationship between smoking and hearing impairment. Only few studies acknowledge the causes of hearing loss due to cigarette smoking and alcohol drinking^24–27^. However, some studies failed to find any association between them^28,29^. Although the underlying mechanisms regarding the effect of smoking on auditory organ is unclear, studies pointed out the mechanisms including direct ototoxicity of nicotine, cochlear ischemia due to increased level of cardoxyhemoglobin, and smoking-mediated increased blood viscosity^30^. Moreover, evidence from many studies have acknowledge the association of increased risk of chronic condition among the vision and hearing impaired individual^31,32^. Of these chronic condition diabetes and hypertension which are the common degenerative diseases among the ageing population have been found to be closely link with ageing-related vision impairment and hearing loss^33,34^. Vision loss among diabetic patient is cause when high blood sugar damages the blood vessel in the retina. Damage blood vessels may leak and swell, resulting in blurry vision or stopping blood flow^35^. Hearing loss among the hypertensive is related to a microcirculatory insufficiency that occurs due to vascular occlusion caused by emboli, haemorrhage or vasospasm^36^. Studies have also found a link between socio-economic and demographic factors such as age, education, living arrangement, and marital status with vision and hearing impairment^19,21,25,30^.

Hearing difficulty is said to affect physical and social functioning, which may further have an impact on behavioural disorders, mood disturbances, and cognitive deficits^13,17,18^. Most of the hearing losses among older adults could be responsive to amplification^37,38^. Although hearing impairment among older adults can be treated using a hearing aid, accessibility and affordability to these aids becomes a challenge for the underprivileged older adults. In India, a considerable proportion of the population lives in rural areas without social and economic security and without access to proper medical care, which leaves them helpless in utilizing hearing aids^39^. Furthermore, various reasons are found to be associated with the under-utilization of hearing aids among older adults population^40^. Archana et al. noted attitude related factors and device-related factors to be most significant for non-use of hearing aids^40^. Vision difficulty is another common ailment among older adults in India. More than 50 million people in India were estimated to have low vision^41^. Uncorrected refractive errors and cataract are the two most significant factors of visual impairment among older adults^41^. Studies have noted a lower level of utilization of eye care services among older adults^42^. Various reasons were attributed to poor utilization of eye care services in India, no importance to eyes^43^, low education attainment, and low income^44^.

This study aimed to estimate the determinants of vision and hearing difficulty and decision to use vision and hearing aids. The analysis was performed under the assumptions that reporting vision and hearing difficulty and decision to use aids for the problem is a sequential decision making process. In our study about 40% and 80% of the older adults did not report any vision and hearing difficulty respectively, and for using vision and hearing aids (as a dependent variable) for them was zero. Thus, these zero observations result to sample selection biased leading to biased parameter estimation if the appropriate statistical model is not adopted. It is found that the two-stage regression model, Tobit model etc. are widely used methods to deal with this kind of data^45^. However, these model is acknowledging as restrictive because they are unable to provide a holistic picture that demonstrates an individual’s underlying sequential decision-making process: whether or not having vision or hearing difficulty (i.e., participant decision) leading to a decision of whether to seek treatment or not (i.e., treatment decision). The present study adopted the Heckman selection model to address the critical drawbacks of the other models^46^. The Heckman model adopted a two-step process that describes individual’s decision of using vision or hearing aids. According to this model, older adults report of having vision or hearing difficulty or not, and then decide whether to use vision or hearing aids or not. Further, to accommodate the heterogeneity (i.e., shared unobserved factors) among older adults and then address the endogeneity (between hearing and vision loss problems and its treatment seeking behaviour) for older adults in India, the model offers a two-step analysis and deals with the zero-sample issue^47^.

In this study we hypothesize that there is no relationship between having vision and hearing difficulty with decision making of using vision or hearing aid for the problem among older adults.

## Data and methods

### Data

The current study employed data from the Building a Knowledge Base on Population Aging in India (BKPAI) survey, which was conducted across seven Indian states in 2011. The survey collected data on several socioeconomic and health aspects of ageing in households with members aged 60 and up. The survey included seven large regionally representative states with higher populations of people aged 60 and up than the national average. The Primary Sampling Units (PSUs) were chosen using the probability proportional to population size (PPS) technique, and elderly households were picked using systematic sampling within each PSU. The northern section of the country is represented by Punjab and Himachal Pradesh, the southern part by Kerala and Tamil Nadu, the eastern part by Orissa and West Bengal, and the western part by Maharashtra. The states were chosen because they had a higher percentage of people aged 60 and up than the national average and represented all of the country's regions. As a result, the survey was deemed nationally representative.

Villages in rural regions and urban wards in urban areas were the primary sample units (PSUs). Each state had a set sample of 1280 elderly households. More information on the sampling technique and sample size may be found in BKPAI's national and state reports from 2011^48^. The effective sample size for the current study was 9541 older individuals aged 60 and up from seven states. The research was carried out in conformity with all applicable laws and regulations. All the methods were applied as per relevant guidelines.

### Outcome variables

There were two outcome variables used in the present study. Firstly, whether older adults were having any difficulty in vision (no or yes) and hearing (no or yes)? Secondly, whether older adults use any aids for self-reported vision and hearing impairment? Spectacles or lenses (no or yes) and hearing aid (no or yes).

### Predictor variables

The predictor variables were categorized according to the previous literature. The predictor variables are as follows; Smoking tobacco (no or yes), chewing tobacco (no or yes), alcohol consumption (no or yes), diabetes (no or yes), hypertension (no or yes), stroke (no or yes), heart disease (no or yes), cataract (no or yes). Diabetes, hypertension, stroke, heart disease and cataract was assed using the question “has a doctor or nurse ever told you that you have Diabetes, hypertension, stroke, heart disease or cataract?”. Further variables were categorized into age (60–69, 70–79 and 80+), gender (men and women), marital status (not in a union and currently in a union), education (not educated, below five years, 6–10 years and 11+ years), working status (no, yes and retired), economic independence (independent, pension and dependent), living arrangement (alone, with the spouse, with children and others), wealth (poor, middle and rich). The wealth index drawn based on the BKPAI survey is based on the following 30 assets and housing characteristics: drinking water source; household electrification; type of toilet facility; cooking fuel; type of house; house ownership; ownership of a bank or post-office account; and ownership of a mattress, a pressure cooker, a chair, a cot/bed, a table, an electric fan, a radio/transistor, a colour television, a black and white television, a sewing machine, a mobile telephone, any landline phone, internet facility, a computer; a refrigerator, a watch or clock, a bicycle, an animal-drawn cart, a motorcycle or scooter, a car, a water pump, a thresher, and a tractor. Religion (Hindu, Muslim, Sikh, and others), caste (Scheduled Caste/Scheduled Tribe (SC/ST) and non-SC/ST), residence (rural and urban) and states (Himachal Pradesh, Punjab, West Bengal, Orrisa, Maharashtra, Kerala, and Tamil Nadu).

### Statistical analysis

Preliminary analysis was carried out using descriptive statistics and bivariate analysis. The Heckprobit selection model, a two-equation model, was also used in the study^49–51^. First, there is a selection model (in this study, referring to “Do you have any of the following difficulties either vision or hearing? (yes or no)”). Secondly, there is an outcome model with a binary outcome (in this study refers to “Do you use any of the following aids either spectacles or lenses or a hearing aid? (yes or no)”). The model provides a two-step analysis and deals with the zero-sample issue, based on which it can accommodate the heterogeneity (i.e., shared unobserved factors) between older adults and then address the endogeneity (between difficulties in vision or hearing and opting for aid for the problem) for older adults in India. When the same independent variables from the selection equation occur in the result equation, the Heckman model is identified^52–54^. Because of the significant multicollinearity, this does not yield precise estimates in the outcome equation; it was advised that at least one independent variable must appear in the selection equation but not in the outcome equation. Statistical significance was defined as a p-value of less than 0.05.

The probit model with sample selection assumes that there exists an underlying relationship^54^:

$${y}_{j}= {x}_{j}{\beta +{u}_{1j}\,\,\,\mathrm{ latent equation}}$$

such that we observe only the binary outcome^54^

$${y}_{i}^{probit}=\left({y}_{j}>0\right)\,\,\,\mathrm{ probit\,\, equation}$$

The dependent variable, however, is not always observed. Rather, the dependent variable for observation j is observed if:

$${y}_{i}^{select}=\left({z}_{j}\gamma +{u}_{2j}\right)>0 \mathrm{selection\,\, equation}$$$$where$$, $${u}_{1}\sim N (0, 1)$$, $${u}_{2}\sim N (0, {\sigma }^{2})$$, $$Corr \left({u}_{1},{u}_{2}\right)=\rho$$.

When $\rho$ ǂ 0, standard probit techniques applied to the first equation yield biased results. Heckprobit provides consistent, asymptotically efficient estimates for all the parameters in such models. The selection equation should have at least one variable that is not in the probit equation for the model to be well identified. The model is otherwise solely identified by its functional form, and the coefficients have no structural meaning.

## Results

**Figure 1:**
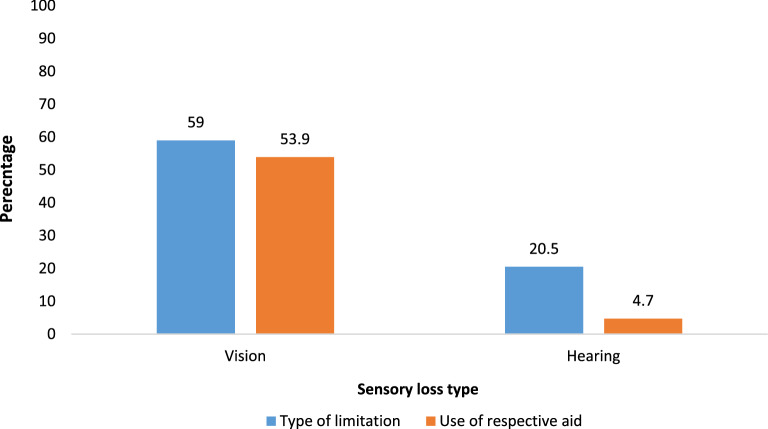
Percentage of older adults suffering from vision and hearing disability and their utilization of visual and hearing aids.

Figure 1 shows that more than half (59%) of the older adults reported vision problems and about 53.9% of them were using visual aids for the same (59% of the older adults in the sample). Moreover, one-fifth of older adults (20.5%) were suffering from hearing difficulty, and about 5% of them were using hearing aids (20.5% of the older adults in the sample). The hearing and vision loss are self-reported.

The socio-demographic profile of older adults was presented in Table 1. About fifteen percent of older adult’s smoke, 22% chewed tobacco, and another 8% consumed alcohol. Around 10%, 21%, 6%, and 13% of older adults were suffering from diabetes, hypertension, heart disease, and cataract, respectively. A majority of older adults belonged to 60–69 years’ age group, 53% were women, and three-fifths of the study population was currently in a union. About half of the older adults had no education and were dependent on pension, 67% were not working, and 70% were living with their children. One-fourth of the study population lived in urban areas.

**Table 1:** Sample distribution of study population, India.

Variables	N	%
**Smoking tobacco**		
No	8085	84.7
Yes	1456	15.3
**Chewing tobacco**		
No	7481	78.4
Yes	2060	21.6
**Alcohol consumption**		
No	8814	92.4
Yes	727	7.6
**Diabetes**		
No	8570	89.8
Yes	971	10.2
**Hypertension**		
No	7520	78.8
Yes	2021	21.2
**Stroke**		
No	9448	99.0
Yes	93	1.0
**Heart disease**		
No	8991	94.2
Yes	550	5.8
**Cataract**		
No	8305	87.1
Yes	1236	13.0
**Age (years)**		
60–69	5891	61.8
70–79	2613	27.4
80+	1036	10.9
**Gender**		
Men	4526	47.4
Women	5015	52.6
**Marital status**		
Not in union	3758	39.4
Currently in union	5783	60.6
**Education**		
No education	4870	51.1
Below 5 years	1955	20.5
6–10 years	2137	22.4
11+ years	578	6.1
**Working status**		
No	6421	67.3
Yes	2310	24.2
Retired	810	8.5
**Economic independence**		
Independent	2178	22.8
Pension	2772	29.1
Dependent pension	4591	48.1
**Living arrangement**		
Alone	561	5.9
With spouse	1523	16.0
With children	6717	70.4
Others	740	7.8
**Wealth status**		
Poor	4367	45.8
Middle	1969	20.6
Rich	3204	33.6
**Religion**		
Hindu	7572	79.4
Muslim	671	7.0
Sikh	898	9.4
Others	400	4.2
**Caste**		
SC/ST	2510	26.3
Non-SC/ST	7031	73.7
**Residence**		
Rural	7044	73.8
Urban	2497	26.2
**State**		
Himachal Pradesh	1470	15.4
Punjab	1354	14.2
West Bengal	1127	11.8
Orissa	1453	15.2
Maharashtra	1379	14.5
Kerala	1356	14.2
Tamil Nadu	1403	14.7

*N:* sample, *%:* percentage, *SC/ST:* scheduled caste/scheduled tribe.

Table 2 found that more than half of the older adults who reported the use of smoking (65%), chewing tobacco (69%), and alcohol consumption (62%) were suffering from a vision problem. Seven in every ten older adults suffering from diabetes (73%), hypertension (74%), stroke (74%), and heart diseases (74%) reported vision problems. Around 61% of women and 54% of working older adults reported a vision problem. Moreover, the use of visual aid was higher among men (57.7%), having 11+ education (84.4%), and rich older adults (76.1%). On the other hand, the hearing problem was more prevalent among older adults who smoke (21.5%), chew tobacco (24.5%), and were suffering from diabetes (21.2%), hypertension (23.6%), stroke (40.4%), and heart disease (26.5%) compared to their counterparts. Around 46 percent of the older adults aged 80+ were suffering from hearing problems. Interestingly, hearing problem (22%) was higher among women, but the use of hearing aid was higher in men (5.1%).

**Table 2:** Bivariate association between background characteristics and vision & hearing disability along with AID seeking behavior among older adults in India.

Background characteristics	Vision disability (%) (N = 9541)	Visual aid (%) (N = 5738)	Hearing disability (%) (N = 9541)	Hearing aid (%) (N = 1872)
**Smoking tobacco**				
No	57.9		20.4	
Yes	65.3		21.5	
**Chewing tobacco**				
No	56.3		19.5	
Yes	69.2		24.5	
**Alcohol consumption**				
No	58.8		20.7	
Yes	62.2		18.7	
**Diabetes**				
No	57.5		20.5	
Yes	72.8		21.2	
**Hypertension**				
No	55.1		19.7	
Yes	73.8		23.6	
**Stroke**				
No	58.9		20.3	
Yes	73.6		40.4	
**Heart disease**				
No	58.1		20.2	
Yes	73.8		26.5	
**Cataract**				
No	54.4		N.A	
Yes	90.6		N.A	
**Age (years)**				
60–69	52.7	55.1	12.5	2.3
70–79	67.0	51.6	28.6	5.9
80+	75.3	54.1	45.6	6.6
**Gender**				
Men	57.3	57.7	18.9	5.1
Women	60.6	50.7	22.0	4.4
**Marital status**				
Not in union	63.8	49.4	26.6	4.5
Currently in union	56.0	57.2	16.6	4.9
**Education**				
No education	59.1	42.3	25.1	3.9
Below 5 years	63.5	52.4	20.6	4.7
6–10 years	53.0	74.7	12.7	4.5
11+ years	66.0	84.4	11.2	20.0
**Working status**				
No	62.1	51.7	23.7	4.6
Yes	53.8	50.8	14.5	4.0
Retired	50.0	85.2	12.6	7.8
**Economic independence**				
Independent	49.5	53.4	14.0	5.1
Pension	66.0	54.5	24.8	4.2
Dependent pension	59.4	53.7	21.1	4.9
**Living arrangement**				
Alone	56.4	43.2	23.7	2.1
With spouse	50.7	50.3	17.1	3.5
With children	60.2	54.3	20.6	5.0
Others	67.5	63.1	24.9	5.7
**Wealth status**				
Poor	58.1	35.2	23.7	3.3
Middle	55.9	56.7	20.5	3.6
Rich	62.3	76.1	16.3	8.4
**Religion**				
Hindu	57.7	49.9	21.2	4.7
Muslim	68.6	59.3	20.1	4.1
Sikh	56.2	71.5	16.5	3.4
Others	73.9	74.0	18.6	7.8
**Caste**				
SC/ST	59.0	40.0	24.7	2.4
Non-SC/ST	59.1	58.8	19.1	5.8
**Residence**				
Rural	59.1	49.8	22.0	4.1
Urban	58.8	65.5	16.6	7.1
**State**				
Himachal Pradesh	48.5	59.0	22.0	5.5
Punjab	58.8	70.2	15.7	5.9
West Bengal	79.7	51.2	32.5	2.8
Orissa	59.8	18.2	26.3	0.2
Maharashtra	65.0	72.1	15.4	8.9
Kerala	72.4	65.8	18.1	7.4
Tamil Nadu	34.1	30.4	15.6	6.4

*%:* percentage, *SC/ST:* scheduled caste/scheduled tribe.

The results from the Heckprobit model for reporting of vision problems and sequential decision making for the use of visual aids are presented in Table 3*.* Results found that older adults who smoked [β = 0.24; CI: 0.15–0.33] and chewed tobacco [β = 0.14; CI: 0.07–0.21] had 0.24 and 0.14 times higher probability to have a vision problem, respectively. Furthermore, older adults who had diabetes [β = 0.25; CI: 0.16–0.34], hypertension [β = 0.31; CI: 0.24–0.38], and those who suffered from heart diseases [β = 0.22; CI: 0.10–0.33] had 0.25, 0.31, and 0.22 times more risk of vision problem, respectively. As expected, those suffering from cataract were highly susceptible to report vision problems. In the age group 70–79 years, the probability of having a vision problem was 0.30 times higher [β = 0.30; CI: 0.23–0.36], and in the age group 80 + years [β = 0.52; CI: 0.42–0.62], it was 0.52 times higher than the referenced category of age 60–69 years. Older adults with 11 + education had 0.18 times higher chances to report vision problems than older adults with no education [β = 0.18; CI: 0.06–0.31], and a similar result was found for using visual aids [β = 0.54; CI: 0.37–0.70]. Rich (0.52 times) older adults had higher probability to use vision aids compared to poor older adults [β = 0.52; CI: 0.42–0.63].

**Table 3:** Heckprobit model for vision disability and using visual aids among older adults in India.

Background characteristics	Outcome equation (vision disability)	Selection equation (visual aid)
**Smoking tobacco**		
No	Ref	
Yes	0.24* (0.15, 0.33)	
**Chewing tobacco**		
No	Ref	
Yes	0.14*(0.07, 0.21)	
**Alcohol consumption**		
No	Ref	
Yes	−0.03 (−0.14, 0.08)	
**Diabetes**		
No	Ref	
Yes	0.25* (0.16, 0.34)	
**Hypertension**		
No	Ref	
Yes	0.31* (0.24, 0.38)	
**Stroke**		
No	Ref	
Yes	−0.03 (−0.31, 0.25)	
**Heart disease**		
No	Ref	
Yes	0.22* (0.1, 0.33)	
**Cataract**		
No	Ref	
Yes	0.94* (0.84, 1.05)	
**Age (years)**		
60–69	Ref	Ref
70–79	0.3* (0.23, 0.36)	−0.11* (−0.19, −0.03)
80+	0.52* (0.42, 0.62)	−0.11 (−0.22, 0)
**Gender**		
Men	Ref	Ref
Women	0.03 (−0.05, 0.1)	0.06 (−0.03, 0.14)
**Marital status**		
Not in union	Ref	Ref
Currently in union	−0.04 (−0.11, 0.04)	0.04 (−0.05, 0.12)
**Education**		
No education	Ref	Ref
Below 5 years	0.06 (−0.02, 0.14)	0.19* (0.1, 0.28)
6–10 years	−0.06 (−0.14, 0.02)	0.58* (0.47, 0.68)
11+ years	0.18* (0.06, 0.31)	0.54* (0.37, 0.7)
**Working status**		
No	Ref	Ref
Yes	0.01 (−0.08, 0.1)	−0.08 (−0.19, 0.03)
Retired	−0.2* (−0.31, −0.09)	0.41* (0.26, 0.57)
**Economic independence**		
Independent	Ref	Ref
Pension	0.26* (0.17, 0.36)	−0.19*(−0.31, −0.07)
Dependent pension	0.13* (0.04, 0.22)	−0.19*(−0.3, −0.07)
**Living arrangement**		
Alone	Ref	Ref
With spouse	−0.2* (−0.34, −0.06)	–0.02 (−0.2, 0.16)
With children	−0.14*(−0.27, −0.02)	−0.02 (−0.17, 0.13)
Others	−0.08 (−0.24, 0.07)	0.04 (−0.14, 0.21)
**Wealth status**		
Poor	Ref	Ref
Middle	−0.05 (−0.13, 0.03)	0.26* (0.16, 0.36)
Rich	0.11* (0.03,0.19)	0.52* (0.42, 0.63)
**Religion**		
Hindu	Ref	Ref
Muslim	−0.02 (−0.13, 0.1)	−0.09 (−0.22, 0.04)
Sikh	−0.16* (−0.29, −0.02)	0.09(−0.08, 0.26)
Others	0.08 (−0.07, 0.23)	0.05(−0.11, 0.22)
**Caste**		
SC/ST	Ref	Ref
Non-SC/ST	0.04 (−0.03, 0.11)	0.13* (0.05, 0.22)
**Residence**		
Rural	Ref	Ref
Urban	0.03 (−0.03, 0.09)	0.05 (−0.02, 0.12)
**State**		
Himachal Pradesh	Ref	Ref
Punjab	0.19* (0.07, 0.32)	0.01 (−0.16, 0.15)
West Bengal	0.73* (0.62, 0.85)	−0.46* (−0.59, −0.33)
Orissa	0.24* (0.14, 0.35)	−0.86*(−0.99, −0.72)
Maharashtra	0.33* (0.22, 0.43)	0.35* (0.21, 0.49)
Kerala	0.25* (0.13, 0.36)	−0.35* (−0.48, −0.22)
Tamil Nadu	−0.26* (−0.36, −0.15)	−0.35* (−0.51, −0.19)
**/athrho**	−0.98* (−1.25, −0.72)	
**rho**	−0.75* (−0.85, −0.62)	
**Wald chi2**	860.92*	
**Censored observation**	3803	
**Uncensored observation**	5738	

*Ref:* reference, *SC/ST:* scheduled caste/scheduled tribe.

*If p < 0.05.

The results from the Heckprobit model for reporting of hearing problems and sequential decision making for the use of hearing aids are presented in Table 4*.* Older adults suffering from diabetes [β = 0.10; CI: 0.00–0.20] and heart diseases [β = 0.19; CI: 0.06–0.31] had 0.10 times and 0.19 times higher probability to report hearing problems, respectively, as compared to those who were not suffering from those diseases. Moreover, older adults currently in a union, having any level of education, and retired from work had lower probability to report hearing problems than their counterparts. On the other hand, older adults with 11 + education [β = 0.60; CI: 0.18–1.02] and living with children [β = 0.47; CI: 0.03–0.97] had 0.60 times and 0.47 times, respectively, higher probability to use hearing aids compared to older adults having no education and living alone.

**Table 4:** Heckprobit model for hearing disability and using hearing aids among older adults in India.

Background characteristics	Outcome equation (hearing disability)	Selection equation (hearing aid)
**Smoking tobacco**		
No	Ref	
Yes	0.08 (−0.03, 0.18)	
**Chewing tobacco**		
No	Ref	
Yes	0.03 (−0.05, 0.11)	
**Alcohol consumption**		
No	Ref	
Yes	0 (−0.13, 0.14)	
**Diabetes**		
No	Ref	
Yes	0.1* (0, 0.2)	
**Hypertension**		
No	Ref	
Yes	0.07 (−0.01, 0.14)	
**Stroke**		
No	Ref	
Yes	0.2 (−0.07, 0.48)	
**Heart disease**		
No	Ref	
Yes	0.19* (0.06, 0.31)	
**Age (years)**		
60–69	Ref	Ref
70–79	0.49* (0.42, 0.56)	0.24 (−0.47, 0.95)
80+	0.91* (0.81, 1)	0.21(−0.9, 1.32)
**Gender**		
Men	Ref	Ref
Women	−0.07 (−0.15, 0.02)	0.08 (−0.18, 0.34)
**Marital status**		
Not in union	Ref	Ref
Currently in union	−0.1* (−0.18, −0.02)	0.18 (−0.07, 0.43)
**Education**		
No education	Ref	Ref
Below 5 years	−0.09* (−0.18, −0.01)	0.07 (−0.2, 0.35)
6–10 years	−0.23* (−0.33, −0.14)	0.17 (−0.17, 0.5)
11 + years	−0.25* (−0.4, −0.1)	0.6* (0.18, 1.02)
**Working status**		
No	Ref	Ref
Yes	−0.15* (−0.25, −0.04)	0.26 (−0.09, 0.61)
Retired	−0.22* (−0.35, −0.08)	0.04 (−0.41, 0.48)
**Economic independence**		
Independent	Ref	Ref
Pension	0.23* (0.12, 0.34)	−0.05 (−0.49, 0.38)
Dependent pension	0.14* (0.03, 0.25)	−0.02 (−0.41, 0.36)
**Living arrangement**		
Alone	Ref	Ref
With spouse	−0.08 (−0.24, 0.07)	0.2 (−0.36, 0.76)
With children	−0.11 (−0.24, 0.03)	0.47* (0.03, 0.97)
Others	0.03 (−0.14, 0.19)	0.5 (−0.1, 1.1)
**Wealth status**		
Poor	Ref	Ref
Middle	−0.05 (−0.14, 0.04)	−0.19 (−0.51, 0.14)
Rich	−0.07 (−0.17, 0.02)	0.11 (−0.15, 0.38)
**Religion**		
Hindu	Ref	Ref
Muslim	−0.06 (−0.2, 0.07)	−0.1 (−0.53, 0.34)
Sikh	0.09 (−0.08, 0.26)	−0.6* (−1.13, −0.07)
Others	0.01 (−0.15, 0.17)	−0.07 (−0.51, 0.37)
**Caste**		
SC/ST	Ref	Ref
Non-SC/ST	−0.07 (−0.15, 0)	0.22 (−0.03, 0.47)
**Residence**		
Rural	Ref	Ref
Urban	−0.03 (−0.1, 0.03)	0.14 (−0.07, 0.34)
**State**		
Himachal Pradesh	Ref	Ref
Punjab	− 0.33* (−0.48, −0.17)	0.37 (−0.07, 0.82)
West Bengal	0.41* (0.29, 0.53)	−0.53* (−0.91, −0.15)
Orissa	0.2* (0.09, 0.32)	−1* (−1.53, −0.48)
Maharashtra	−0.14* (−0.26, −0.01)	0.2 (−0.14, 0.53)
Kerala	−0.05 (−0.18, 0.07)	−0.17 (−0.51, 0.18)
Tamil Nadu	−0.05 (−0.17, 0.08)	−0.03 (−0.41, 0.34)
**/athrho**	−0.52* (−1.83, −0.10)	
**rho**	−0.48* (−0.95, 0.66)	
**Wald chi2**	84.43*	
**Censored observation**	7669	
**Uncensored observation**	1872	

*Ref:* reference, *SC/ST:* scheduled caste/scheduled tribe.

*If p < 0.05.

## Discussion

Vision and hearing disabilities are particularly prominent problems in the aging population. While vision difficulty harms a person’s physical senses of the surrounding world, hearing difficulty diminishes a person’s mode of social interactions and can lead to social isolation. Deteriorations in vision and hearing are associated with reduced quality of life, increased physical difficulty, imbalance, falls, hip fracture, and mortality^55–57^. A longitudinal study in UK provides evidence that hearing impairment among older adults is associated with loneliness, social isolation and the effect on cognitive function^58^. However, the rate of cognitive decline can be delayed or arrested if treatment is provided as earlier as possible with the help of hearing aid device^17,58^. The present study tries to address the issue of various factors associated with vision and hearing difficulty among older adults in India. The study reported that vision difficulty was a prominent difficulty among older adults than hearing difficulty. Various socio-economic, demographic, and behavioural risk factors were found to be associated with vision and hearing difficulty among older adults. We found a high proportion of older adults who reported having vision and hearing difficulty were also smoking, chewing tobacco, drinking alcohol, and suffering from chronic diseases. Also, the proportion of using vision and hearing aid among older adults varies by gender, level of education, and economic status.

Tobacco smoking has long been known as a significant risk factor for many chronic diseases; the effect of smoking on vision loss is not well recognized. Our results from the heckprobit model provide evidence that older adults who use smoked and chewed tobacco were more likely to report having vision difficulty, and the results are consistent with the previous results^19^. Previous literature has acknowledged that current smokers with cataract were more likely to suffer visual loss than non-smokers with cataracts^20^. Age-related macular degeneration (AMD) is the main cause of vision difficulty for older adults, although it does not cause complete blindness to a person, it can lead to loss of central vision, which happens very slowly in life. Epidemiological evidence suggests that smoking is a strong causality for age-related macular degeneration, that may promote ischemia, hypoxia, and micro-infractions and reduce choroidal blood flow in the eye, all of which could increase the predisposition of the macula to degenerative changes^59,60^. Our analysis also indicates that difficulty in vision was more likely to occur in older adults with a chronic disease like diabetes, hypertension, and heart disease compared with people without chronic disease, which is consistent with the previous studies conducted in Australia and America^22,55^. The possible explanation is that physical inactivity among individuals has been identified as the main factor attributed to the development of non-communicable disease^32^. Vision-impaired individuals are more likely to be physically inactive due to the fear of falls, which then puts them at a higher risk of developing chronic disease.^61^ Another reason may be related to eating unhealthy food as vision-impaired individuals may face difficulty in choosing healthy food if they cannot read the food nutrition labels properly^62^.

In terms of living arrangements, it was observed that older adults living alone were more likely to report having a vision difficulty as compare to those living with spouses or children. Previous literature suggests that older people living alone are considered as vulnerable as they were characterized by difficult living condition, lack of social support, high poverty, experience greater challenges in daily activities, and at greater risk of developing any chronic disease due to lifestyle factors^63^. Kharicha et al. mentioned that the difficulty with instrumental activities of daily living among older adults living alone might be related to the risk of chronic diseases like glaucoma, cataract, and arthritis^64^. Literate older adults from better household economic status were more likely to use visual aid as compared to those from poor economic status. Previous studies have acknowledged the use of visual aid, which includes spectacles increased with increasing socioeconomic status and possibly associated with the increased level of education^65^. However, use of spectacles in the rural areas remains low due to the cost of spectacles and low educational level in the rural areas^66,67^, users were found to be more common among the literates and employed people^64,67^.

Older adults with chronic diseases like diabetes and heart disease were found to be associated with having a hearing difficulty, which is consistent with our findings^33,55^. Study shows that peripheral nerve injury and presence of albuminuria among the patients with type 2 diabetes was associated with hearing loss^33,68^. Furthermore, older adults with higher levels of education and living with children were more likely to use a hearing aid. The possible explanation could be due to the indirect effect, as education is closely related to income, and the high cost was one of the key reasons for not using of hearing aid^69,70^, and on the other hand, older adults living with their children usually get their financial support and have access to better health care^71^.

The study had certain limitation. Firstly, the vision and hearing difficulty were self-reported in nature. Secondly, due to the binary response of hearing and visual difficulty, we cannot assess the types of hearing and visual difficulty which are the main contributors of these difficulty. However, apart from all these limitations the present paper presents the results which are quite important from policy point of view.

## Conclusion

Our study has shown that a higher percentage of older adults have vision difficulty than hearing difficulty; moreover, the treatment-seeking for vision difficulty was much higher among older adults than treatment-seeking for hearing difficulty. Lifestyle factors (smoking tobacco and chewing tobacco) significantly affect hearing and vision difficulty; various chronic diseases were also found to be associated with high levels of hearing and vision difficulty among older adults. Hearing and vision difficulty, along with the sequential treatment for these ailments, were significantly affected by various socio-economic characteristics. The use of hearing and visual aids was lower among poor older adults, older adults from Scheduled Caste, and older adults in rural areas. It can be assumed that hearing and vision aids were significantly affected by their cost as the sequential treatment for hearing and vision difficulty was lower among poor older adults. Based on our findings, we recommend that additional attention shall be given to understand the strategies that may advocate a higher use for hearing aids among older adults. Although a well-planned health structure is in place in India, we suggest that this system of health structure shall be re-examined to establish and integrate the various needs of older adults. There is a need to integrate public health approaches into interventions for older adults with hearing and vision impairments.
